# The relationship between first-level leadership and inner-context and implementation outcomes in behavioral health: a scoping review

**DOI:** 10.1186/s13012-021-01104-4

**Published:** 2021-07-06

**Authors:** Rosemary D. Meza, Noah S. Triplett, Grace S. Woodard, Prerna Martin, Alya N. Khairuzzaman, Gabrielle Jamora, Shannon Dorsey

**Affiliations:** Department of Psychology, University of Washington, Guthrie Hall 119A, Box 351525, Seattle, WA 98195 USA

**Keywords:** First-level leadership, Implementation outcomes, Implementation determinants, Inner-context outcomes, Behavioral health

## Abstract

**Background:**

First-level leadership is uniquely positioned to support evidence-based practice (EBP) implementation for behavioral health due to first-level leaders’ access to and relationship with service providers. First-level leaders are individuals who directly supervise and manage frontline employees who do not manage others. However, first-level leadership is underrepresented in existing reviews of the impact of leadership on EBP implementation. This review describes the relationship between first-level leadership and implementation determinants and outcomes.

**Methods:**

A scoping review was performed to synthesize the literature on the relationship between first-level leadership and inner-context and implementation outcomes. A literature search was conducted in PubMed, Eric, PsycINFO, CINAHL, Scopus, and Web of Science. To be eligible, studies had to examine first-level leadership, be conducted in settings providing behavioral health services, and examine the relationship between first-level leadership and an implementation or inner-context outcome. Data extraction and synthesis were performed to describe study characteristics, leader-outcome relationships, and overlap in leadership frameworks.

**Results:**

Twenty-one records met our inclusion criteria. Studies primarily relied on observational designs and were often cross-sectional. Studies more often examined general leadership rather than leadership strategically focused on EBP implementation (i.e., strategic implementation leadership). Our findings suggest that several forms of first-level leadership are inconsistently related to a broad set of implementation determinants, with infrequent examination of specific implementation outcomes. The broad set of implementation determinants studied, limited number of replications, and inconsistent findings have resulted in sparse evidence for any specific leadership-outcome relationship. The greatest accumulation of evidence exists for general leadership’s positive relationship with providers’ EBP attitudes, most notably in the form of transformational leadership. This was followed by evidence for strategic implementation leadership facilitating general implementation. Our synthesis revealed moderate conceptual overlap of strategic implementation leadership behaviors described in the theory of implementation leadership and theory of middle managers’ role in implementation.

**Conclusions:**

Our findings suggest that first-level leadership may play an important role in shaping implementation determinants and outcomes, but consistent empirical support is sparse and confidence dampened by methodological issues. To advance the field, we need studies that adopt stronger methodological rigor, address the conceptual overlap in leadership frameworks, examine a broader set of implementation outcomes, and examine conditions under which leadership impacts implementation.

**Trial registration:**

This review was not registered.

**Supplementary Information:**

The online version contains supplementary material available at 10.1186/s13012-021-01104-4.

Contributions to the literature

- This is the first scoping review to explore how first-level leadership shapes implementation determinants and outcomes for behavioral health services.
- The positive relationship between first-level transformational leadership and providers’ EBP attitudes was a primary focus of studies; however, the evidence linking provider attitudes to implementation outcomes is, at best, mixed.
- There was very limited evidence linking any form of first-level leadership to a specific implementation outcome and findings were inconsistent.
- Our qualitative synthesis suggests that there is significant conceptual overlap in behaviors described in the theories of implementation leadership and middle managers’ role in implementation, highlighting the need to disentangle how these strategic forms of implementation leadership uniquely relate to EBP implementation.
- We offer conceptual and methodological guidance for the field to advance our understanding of whether and how first-level leadership supports EBP implementation.

## Introduction

Despite advances in evidence-based practices (EBPs) to treat mental and behavioral health problems [1], efforts to transfer them from laboratory to public settings are often unsuccessful [2, 3]. Implementation frameworks suggest that leadership may function as a mechanism for improving EBP implementation [4–7]. Empirical studies support this theoretical link between leadership and the innovation implementation in healthcare settings [8–10]. While there is no universally adopted leadership definition, conceptualizations generally reflect a process of intentional efforts by an individual to motivate, influence, and enable a person or group of people with the aim of impacting group or organizational outcomes [11–13]. Leadership across organizational levels—from top leaders who establish organizational policies and practices [14] to first-level leaders who directly supervise employees providing direct services—and their alignment with one another influence the success of implementation [15, 16]. Achieving organizational effectiveness, including effectiveness in EBP implementation, is complex and hinges on coordination across levels of leadership [16, 17]. Organizational leadership at the first-level may be particularly influential in supporting EBP implementation due to leaders’ access to and direct relationships with service providers. For instance, first-level leadership may function to support or hinder the realization of top-level leaders’ organizational policies that are favorable for EBP implementation. Conversely, first-level leadership may mitigate potential negative impacts of organizational policies instituted by top leaders that would hinder implementation.

First-level leaders are individuals who directly supervise and manage frontline employees who do not manage others [18]. In the context of behavioral health service delivery, first-level leaders tend to include clinical supervisors, program managers, and team leaders who supervise direct providers. Leaders at this lowest level enact their leadership influence through direct interaction with frontline employees, making decisions that concern day-to-day work, anticipating and solving current problems, and using practical judgment to address ongoing problems [19]. They engage and inspire staff at the frontline [17]. In the context of implementation, first-level leadership is theorized to improve general and strategic cultures and climates, provider attitudes, and implementation outcomes [4, 16, 20].

There have been calls for leadership to be studied within a particular organizational level and context because the antecedents, consequences, and dynamics of leadership change as a function of organizational level, structure, and complexity [19]. The role of first-level leadership differs from leadership at higher organizational levels. At the highest level, leaders are responsible for establishing a comprehensive direction for the organization, creating organizational policy, and crafting organizational strategy [19]. Mid-level leaders engage in coordination of multiple subunits, manage less directly, and are responsible for establishing operational practices and policies [19]. The success of innovation implementation relies on numerous complex factors, some of which include training, teaching and feedback, time requirements, reimbursement, resource allocation, and workflow integration [4, 5]. Leaders across organizational levels are differentially positioned to impact these factors, which has implications for their influence on implementation. Given the distinct responsibilities of first-level leaders focused on day-to-day decisions and problem solving, synthesis of the literature is needed to understand *how* first-level leadership influences implementation and *what* implementation determinants and outcomes they impact.

Empirical research examining leadership and training programs to develop effective leadership have lagged in behavioral health relative to health broadly. The number of studies examining the role of leadership in behavioral health innovation implementation has not matched that of health innovations broadly [8]. Moreover, first-level leaders within behavioral health tend to be promoted internally due to their effectiveness as providers, thus are often unprepared and lacking formal leadership training [21, 22]. While leadership training is a continued need in healthcare broadly, many examples of formal training programs have existed in medicine for upwards of 20 years [23–26]. Efforts to train leaders in behavioral health, including those with a focus on EBP implementation, are limited (e.g., [27, 28]). Synthesis of the literature is needed to understand the type of leadership styles and behaviors those in behavioral health draw on to support implementation. Further, implementation determinants and outcomes are theorized to interact with innovation characteristics [4, 29]. Behavioral health has followed the lead of evidence-based medicine in using research evidence to standardize healthcare [30] yielding an abundance of beneficial EBPs. Attending to and measuring delivery of EBPs is one important method by which leaders can impact EBT delivery [16]. However, monitoring and evaluating the content and dose of complex behavioral health interventions is difficult [31]. Efforts to standardize delivery and maintain quality have included detailed treatment manuals, training, certification requirements, and fidelity criteria [32], yet modification of behavioral health interventions is common [33] and direct observation to monitor and support delivery is often unfeasible [34]. Further, many treatments are based on principles of practice rather than prescribed strategies (e.g., [35–37]) posing additional challenges to defining and monitoring their adoption, fidelity, and sustained use. These characteristics of behavioral health interventions may shape which first-level leadership behaviors are most effective in impacting implementation and the degree to which first-level leadership is able to impact implementation. For instance, task-oriented leadership behaviors such as planning and monitoring performance [38] may be more effective in ensuring behavioral health interventions are adopted and used with high fidelity than change-oriented behaviors primarily concerned with providing vision and encouraging innovation [38].

First-level leaders may influence implementation through leadership that aims to impact performance outcomes generally (i.e., general leadership) and through leadership that is strategically focused on influencing implementation outcomes (i.e., strategic implementation leadership). The full range leadership model, one of the most studied models of general leadership, describes three primary forms of leadership, transformational, transactional, and laissez-faire leadership [39]. Transformational leadership describes how leaders inspire and motivate employees to perform beyond expectations. Transactional leadership functions through reinforcement and exchanges, where leaders reward employees who fulfill expectations [39–41]. Passive-avoidant leadership is a style of non-leadership [42], characterized as taking a “hands off” approach by altogether avoiding making decisions or managing employees [43]. A recent systematic review found that managers engage in behaviors that are consistent with transformational and transactional approaches to support research use by clinical staff in nursing and allied health professionals [44].

Leadership that is strategically focused on EBP implementation is theorized to promote a positive climate for implementation and, in turn, foster positive attitudes toward EBPs that support implementation [45]. The implementation leadership theory [46] and theory of middle managers’ role in implementation [47, 48] describe leadership behaviors that strategically focus on implementation among first-level leaders. Implementation leadership describes first-level leadership that proactively anticipates and addresses implementation challenges, demonstrates a deep understanding of EBP implementation, perseveres through implementation challenges, and supports providers to adopt and use EBPs [46]. The theory of middle managers’ role in implementation is based on review and research on both first-level leaders and mid-level managers [47–49]. It describes middle manager commitment to innovation implementation operationalized as four ways that managers can demonstrate their commitment for innovation implementation: obtaining and diffusing information about an innovation, adapting information and the innovation, mediating between strategy and day-to-day activities, and selling innovation implementation. In healthcare settings, there is some evidence suggesting that middle manager commitment to implementation—in conjunction with executive support for implementation and access to human resources administration—is associated with implementation effectiveness [50].

### Rationale for the scoping review

Our overarching goal is to synthesize the literature examining how first-level leadership impacts implementation; therefore, we focus on implementation outcomes and determinants. First-level leadership, being situated within the organization’s inner-context, is most suited to impact determinants within the inner-context as described in the Exploration, Preparation, Implementation, and Sustainment (EPIS) framework [4, 29]. These determinants include organizational characteristics, quality and fidelity monitoring and support, organizational staffing processes, and individual adopter characteristics. Similar to the general and strategic focus of leadership, implementation determinants in the inner-context can also be general and strategic. For instance, organizational cultures and climates reflect general organizational characteristics while implementation climate is an organizational characteristic strategically focused on implementation. We focus on inner-context outcomes in addition to implementation outcomes because many inner-context factors function as determinants of implementation outcomes [51, 52].

### Current study

This scoping review aimed to summarize the existing research examining how first-level leadership relates to inner-context and implementation outcomes for behavioral health services. We aimed to clarify key concepts around how leadership has been defined and studied, identify gaps in the knowledge base, and report on the types of evidence that have informed this field [53]. We also aimed to identify future directions to guide this body of research.

## Method

**Fig. 1:**
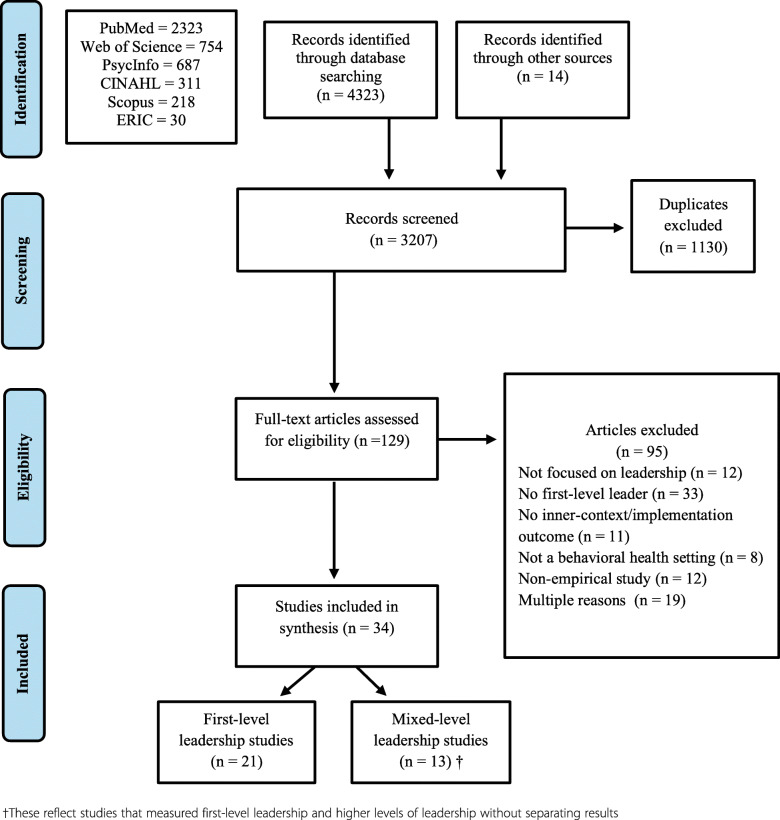
PRISMA Flow Diagram

We followed Arksey and Malley’s [54] methodological framework for scoping reviews. A scoping review was conducted due to the broad, emerging state of literature that cannot be subject to the narrow systematic review criteria due to its heterogenous nature [55]. We followed the preferred reporting items for systematic reviews and meta-analyses extension for scoping reviews (PRISMA-ScR) to enhance transparency. There is no registered review protocol. Additional file 1 includes the PRISMA-ScR checklist and Fig. 1 contains the PRISMA diagram.

### Search strategy

We developed a comprehensive search strategy with a health sciences librarian. To ensure the most relevant articles were identified, we reviewed the title, abstract, and keywords from preselected articles to generate a list of appropriate search terms. Subject headings and keywords were identified and categorized into the following groups (1) implementation; (2) leader, supervisor, manager, or other synonym; (3) intervention; and (4) mental and behavioral health services (see Additional file 2 for search terms). The search was conducted in June 2019 using the following databases: PubMed, Eric, PsycINFO, CINAHL, Scopus, and Web of Science. We also examined the reference lists of studies to identify additional articles. Two additional studies were identified through the Implementation Science journal between the initial search and February 2020.

### Study inclusion and exclusion criteria

Studies were included if they (1) examined the leadership of first-level leaders; (2) were conducted in settings providing behavioral health services; and (3) examined the influence of first-level leadership on an implementation outcome or inner-context outcome based on definitions of inner-context outcomes in the EPIS framework [29] (see Table 6) and implementation outcomes defined by Proctor and colleagues [63]. Studies that measured perceptions of “implementation” as an outcome, which we term “general implementation,” were included if their measurement of implementation conceptually aligned with one or more implementation outcomes [63] (e.g., adoption and fidelity). Studies were excluded if leadership could not be clearly attributed to a first-level leader such as using the term “leader,” “manager,” or “management” without identifying their responsibility for direct supervision of front-line staff. Studies were considered to be providing behavioral health services if they described primarily delivering mental and behavioral health treatments (e.g., addiction health services, mental health services). Studies could be situated in settings that provide a variety of services (e.g., hospitals, human service agencies) if they were examining the delivery of behavioral health services. Review articles without original data, non-empirical studies, and study protocols were excluded. Articles were limited to those published in peer-reviewed journals in English. No restrictions were imposed on publication date, study design, or length of follow-up.

### Study selection and quality assessment

Five reviewers (RM, NST, GSW, AK, GJ) assessed studies independently to determine inclusion status. Titles and abstracts were independently assessed by two reviewers, and each pair of reviewers met to discuss discrepancies. When necessary, consensus was reached with a third reviewer. This process was repeated for full-text review. Consistent with the guidance on conducting scoping reviews, we did not assess the methodological quality or risk of bias of the included articles [53]. Our goal was to provide an overview of the existing evidence base regardless of study quality.

### Data extraction

A draft charting table was developed prior to extraction to capture study characteristics and key findings. The charting table was refined as reviewers trialed the extraction on a small sample of articles. Pairs of reviewers extracted data from each article and discussed discrepancies until they reached consensus. When necessary, a third reviewer was consulted to reach consensus. The extracted data items are listed in Table 1. The extracted items captured first-level leaders’ behaviors, characteristics and/or leadership style examined, inner-context and/or implementation outcomes studied, study characteristics, and key findings.

**Table 1:** Data extraction

Information extracted	Description
Author	List of authors
Year	Publication year
Title	Study title
Country	Country where the study was conducted
Setting	Physical location where the study was conducted (e.g., mental health agency)
Design	Study design as reported or inferred from study methods (e.g., observational, experimental)
Study methodology	Study methodology (qualitative, quantitative, mixed methods)
Data sources	Types of instruments used (e.g., survey, interview)
Measures	Names of measures used and respondents
Construct validity	Whether or not measures had established construct validity
EBP or clinical innovation	EBP or clinical innovation being implemented
Phase of implementation	Stage of the EBP implementation (exploration, preparation, implementation, sustainment, multiple, no active implementation, or not reported)
Leader role	Level of leadership included and leaders’ role in supporting EBP delivery or implementation
First-level leadership	Leadership style, behaviors or characteristics examined
Inner-context outcomes	EPIS inner-context factors examined
Implementation outcomes	Implementation outcomes examined
Level of analysis	Level at which studies measured and analyzed leadership and inner-context and implementation outcomes (individual, team, organization)
Results	Description of the nature of the associations between leadership and inner-context and implementation outcomes

*EBP* evidence-based practice, *EPIS* exploration, preparation, implementation, sustainment framework

### Data synthesis

Synthesis involved quantitative analysis (e.g., descriptive statistics) of study characteristics and qualitative analysis of the leader behaviors and characteristics measured in included studies. We categorized inner-context outcomes based on the original and expanded EPIS framework [4, 29]. We also categorized findings related to implementation outcomes based on Proctor and colleagues [63] framework.

We calculated frequencies to describe the number of studies examining the relationship between leadership and inner-context outcomes. We provide a descriptive summary of the relationship between leadership styles and inner-context and implementation outcomes reported. To do this, we coded results to reflect the direction of the relationship (i.e., positive, negative, or non-significant) reported in studies. A single study could yield multiple discrepant results (i.e., positive, negative, and/or non-significant) if they only measured subscales of a construct (e.g., subscales of transformational leadership). However, when studies reported total scores, we report those associations. The coded relationships include direct associations and indirect mediated associations between leadership and inner-context or implementation outcomes.

We used a deductive approach to code leader behaviors and characteristics in studies that qualitatively measured leadership. We operationalized leader behaviors as actions a leader engages in that may influence the implementation process (e.g., provision of implementation prescriptions, empowering staff) and operationalized leader characteristics as qualities, perceptions, or traits of a leader that may influence the process of implementation (e.g., enthusiasm, attitudes toward EBPs), adapted from Moullin and colleagues [29]. The codebook was developed by first incorporating the leadership styles included in this review’s quantitative studies (e.g., transformational, implementation leadership). Then, we referenced reviews of leadership styles and leader behaviors from the organizational behavior, leadership, and middle manager literature to incorporate other styles of leadership not captured in this review’s quantitative studies (e.g., EBP champion, authentic leadership) [49, 64–67]. When a leader behavior or characteristic did not fit with an existing leadership construct, it was included as a standalone code. Leader behaviors could be coded as reflecting more than one leadership style or framework (e.g., implementation leadership and middle managers’ implementation roles). The percent of overlapping codes were calculated to identify construct overlap in the existing frameworks. All data synthesis was completed by two authors and a third author was consulted as needed.

## Results

The search yielded a total of 4337 articles. After excluding duplicates, 3207 titles and abstracts were reviewed for inclusion. Among those, 129 articles progressed to full-text review and 21 met criteria for data abstraction (Fig. 1). Thirteen additional articles met criteria for abstraction [68–80], but combined their measurement of first-level and higher levels of leadership limiting conclusions about how first-level leadership specifically is related to implementation. Data were abstracted from these articles, but their results are not combined with the 21 primary articles and are listed in the supplemental materials for comparison (additional file 3 Tables 9–12).

### Study characteristics

#### Study methods

Tables 2 and 3 describe the included studies and study characteristics. Studies were most commonly conducted in mental health agencies (81%) and set in the USA (79%). Most used an observational design (90%) and were cross-sectional (57%). Methods included quantitative (57%), qualitative (29%), and mixed methods (14%). Most studies examined the relationship between leadership and an inner-context or implementation outcome while implementing a clinical innovation (62%). Studies were mainly conducted during the implementation (52%) phase.

**Table 2:** Summary of included studies (*N* = 21)

First author	Leadership type(s)	Inner-context outcomes	IS outcomes	Design	Setting	IS phase	Key leadership findings
Aarons 2006 [81]	TfL, TrL	IC	N/a	Obs	CMH	NI	TrL and TfL positively predicted providers' EBPA.
Aarons et al. 2012 [82]	TfL	OC, IC; L	N/a	Exp	CW	I	During EBP implementation, TfL positively predicted InnCli, and InnCli positively predicted providers' EBPA. When delivering services as usual, LMX mediated the relation between leaders' TfL and InnCli.
Aarons et al. 2015 [28]	TfL	OC	N/a	Obs	MH	NI	OrgCul suffered more where leaders rated their TfL more positively than providers rated them, in contrast to where leaders rated themselves lower than providers. OrgCul tended to be better when providers and leaders agreed the leaders’ TfL was high than when they agreed it was low.
Aarons et al. 2016 [83]	TfL, TrL; PaL, LB	N/a	Sus	Obs	CMH, CW, Oth	Sus	TfL positively predicted sustainment, and PaL negatively predicted sustainment. Leaders’ ongoing championing of EBP and practical support for providers facilitated sustainment.
Aarons et al. 2017 [45]	IL	OC	N/a	Obs	MH	NI	OrgCli of involvement and performance feedback were highest when leaders rated their IL low and providers rated leaders’ IL high. Involvement climate did not differ when leaders and providers agreed that IL was strong compared with when they agreed it was weak. Performance feedback climate was higher when leaders and providers agreed that IL was strong.
Brimhall et al. 2016 [84]	TfL	OC, IC	N/a	Obs	CMH	NI	Greater TfL indirectly influenced providers' perceptions of EBPs as less burdensome through higher EmpCli and lower DemoCli.
Bunger et al., 2019 [65]	LB	OC	N/a	Obs	CW	I	Leaders’ activities influenced aspects of ImpCli including conveying expectations, providing support, and rarely rewarding implementation. Leaders conveyed expectations through diffusing information, synthesizing information, mediating between agency strategy and day-to-day activities, and selling implementation. Leaders supported implementation through diffusing, synthesizing, and mediating. They conveyed rewards through diffusing.
Corrigan et al. 2002 [85]	TfL, TrL, PaL	OC, IC	N/a	Obs	H, MH	NI	TfL was positively associated with transformational OrgCul. LfL and passive MBE were negatively associated with a TrC. TfL was positively associated with TrC based on leader report, but negatively associated with TrC based on provider report. Passive MBE was positively associated with TrC based on provider report. TfL was negatively associated with burnout among providers and leaders.
Fenwick et al. 2018 [86]	TfL	IC, F	N/a	Obs	CMH	NI	TfL and LMX positively predicted providers' attitudes toward feedback. LMX mediated the relation between TfL and providers’ attitudes toward feedback.
Fleury et al. 2014 [87]	LB	N/a	GI	Obs	MH, Oth	P, I	Inaccessibility of leaders, leader turnover, and leaders’ poor communication were barriers to implementation.
Green et al. 2014 [88]	TfL	OC	N/a	Obs	CMH	NI	Leaders’ TfL positively predicted EmpCli.
Guerrero et al. 2014 [89]	LC	N/a	A	Obs	AH	NR	Leaders’ EBPA and readiness-for-change attributes positively predicted implementation of contingency management treatment. Leaders’ openness towards EBPs positively predicted implementation of medication-assisted treatment.*
Guerrero et al. 2020 [15]	IL	IC	A	Obs	AH	I	IL was positively associated with provider's EBPA. IL did not mediate the relation among top leaders’ TfL and A.
Mancini et al. 2009 [90]	LB	N/a	GI	Obs	H, MH	I	Leaders’ failure to empower staff, poor organizational skills, poor management of internal dynamics and workload, and turnover were barriers to high-F implementation. Leaders understanding of the model, effective management of team dynamics, holding staff accountable, advocating on behalf of provider teams, empowering staff, conveying a sense of mission to the provider team, and equitably distributing the workload facilitated implementation.
Moser et al. 2005 [91]	LB, LC	N/a	GI	Obs	MH	P, I	Leaders’ turnover, lack of familiarity with the intervention, and lack of investment in implementation were barriers to implementation. Leader familiarity with the intervention facilitated implementation.
Powell et al. 2017 [92]	TfL, IL	IC	N/a	Obs	CMH	I, Sus, NI	TfL-idealized influence positively predicted providers’ knowledge of EBP. TfL-individual consideration negatively predicted providers' EBPA. Proactive IL positively predicted providers’ EBPA. Perseverant IL negatively predicted providers’ EBPA.
Rapp et al. 2010 [93]	LB	N/a	GI	Obs	MH	I	Leader behaviors were the greatest barrier to implementation, including: not setting expectations; only providing consultation on service-delivery when challenges arose; lacking prescriptions or structure to providers' practice; being overly conflict-avoidant; lacking meaningful feedback for providers; having only superficial knowledge of clinician practice; relying on coaxing and persuasion with no consequences for poor performance; poorly leading group supervision, which was dominated by administrative tasks; lacking follow-through; having competing responsibilities; lacking knowledge of EBP skills and feeling inadequate at supervising practice.
Savill et al. 2018 [94]	LB	N/a	GI	Obs	MH, SMH	I	Leaders facilitated implementation by working to incorporate EBP procedures into existing workflows (i.e., assessment checklists and forms) and meeting regularly with senior administrators and staff to monitor and troubleshoot implementation difficulties.
Van Erp et al. 2007 [95]	LB, LC	N/a	GI	Obs	MH	I	Lack of time for leaders to manage the intervention was a barrier to implementation. Leaders' strong personal commitment demonstrated by their dedication and enthusiasm to implement the intervention facilitated implementation.
Van Erp et al. 2009 [96]	LB	N/a	GI	Obs	H, MH	I	Leaders’ inability to administer the implementation process and to realize necessary conditions for implementation were barriers to implementation. Leader motivation facilitated implementation.
Williams et al. 2020 [97]	IL	OC	A	Quas	CMH	Mul	Increases in IL had a significant indirect effect on increases in clinicians’ EBP use via improvement in EBP ImpCli.

*A* adoption, *AH* addiction health agencies, *CMH* child mental health agencies, *CW* child welfare, *DemoCli* demoralizing climate, *EBP* evidence-based practice, *EBPA* evidence-based practice attitudes, *EmpCli* empowering climate, *Exp* experimental, *F* fidelity, *GI*, general implementation, *H* hospital, *I* implementation, *IC* individual characteristics, *IL* implementation leadership, *ImpCli* implementation climate, *InnCli* innovation climate, *L* leadership, *LB* leader behaviors, *LC* leader characteristics, *LfL* laissez-faire leadership, *LMX* leader-member exchange, *MBE* management by exception, *MH* mental health agencies, *Mul* multiple phases-unspecified, *N/A* not applicable, *NI* no active implementation, *NR* not reported, *Obs* observational, *OC* organizational characteristics, *OrgCli* organizational climate, *OrgCul* organizational culture, *Oth* other, *P* preparation, *PaL* passive-avoidant leadership, *Quas* quasi-experimental, *SMH* school-based mental health, *Sus* sustainment, *TfL* transformational leadership, *TrC* transactional culture, *TrL* transactional leadership

**Table 3:** Study characteristics (*N* = 21)

Characteristics	*N* (%)
Setting^a^	
Child welfare	3 (14%)
Mental health agencies	17 (81%)
Substance use agencies	2 (10%)
Hospital	3 (14%)
Other	3 (14%)
Country	
Canada	1 (5%)
Netherlands	2 (10%)
United States	18 (86%)
Design	
Observational	19 (90%)
Quasi-/Experimental	2 (10%)
Method	
Quantitative	12 (57%)
Qualitative	6 (29%)
Mixed	3 (14%)
Data source^a^	
Survey	15 (71%)
Interview	8 (38%)
Focus groups	3 (14%)
Field notes	3 (14%)
Record reviews	3 (14%)
Used a leadership questionnaire	13 (62%)
Used a validated questionnaire (*n* = 13)	12 (92%)
Used a partially validated questionnaire (*n* = 13)	1 (8%)
Used an inner-context and/or implementation outcome questionnaire	15 (71%)
Used a validated questionnaire (*n* = 15)	11 (73%)
Used a partially validated questionnaire (*n* = 15)	11 (73%)
Used an unvalidated questionnaire (*n* = 15)	1 (7%)
Implemented a clinical innovation	13 (62%)
Phase of implementation^a^	
Preparation	2 (10%)
Implementation	11 (52%)
Sustainment	2 (10%)
Multiple phases (*unspecified*)	1 (5%)
No active implementation	8 (38%)
Not reported	1 (5%)
Leadership examined^a^	
General leadership	16 (76%)
Transformational leadership	14 (67%)
Transactional leadership	6 (29%)
Passive-avoidant leadership	4 (19%)
Strategic leadership	12 (57%)
Implementation leadership	11 (52%)
Middle-managers’ roles in implementation^b^	6 (29%)
EBP champion^b^	2 (10%)
Leader behaviors^b^ (uncategorized)	5 (24%)
Leader characteristics^b^ (uncategorized)	1 (5%)
Inner-context outcomes^a^	12 (57%)
Organizational characteristics^a^	8 (38%)
Organizational climate	3 (14%)
Organizational culture	2 (10%)
Implementation climate	2 (10%)
Innovation climate	1 (5%)
Individual characteristics^a^	8 (38%)
Attitudes towards EBP	6 (29%)
Knowledge of EBP	1 (5%)
Burnout	1 (5%)
Implementation outcomes	11 (52%)
Adoption	3 (14%)
Fidelity	1 (5%)
General implementation	6 (29%)
Sustainment	1 (5%)

*N* = 21 unless specified otherwise

^a^Responses are not mutually exclusive

^b^The studies reporting these leadership roles and behaviors were all qualitative studies

Thirteen studies (62%) used a questionnaire to measure first-level leadership and of those, 92% used a leadership questionnaire with established construct validity. Fifteen studies (71%) used a questionnaire to measure either or both inner-context and implementation outcomes. Of those fifteen studies, 73% relied fully on an instrument with established construct validity. Of the five studies measuring a specific implementation outcome, 60% relied on self-report from organizational members. Of the 13 quantitative studies, most (69%) relied on a single respondent for independent and dependent measures.

Table 4 summarizes the level of analysis for leadership and outcomes. Studies tended to analyze perceptions of first-level leadership at the team (43%) and organization-level (43%). Inner-context outcomes were mainly studied at the individual-level (43%) while implementation outcomes were primarily studied at the organization-level (43%).

**Table 4:** Study level of analysis

Level	Leadership	Inner-context	Implementation
Individual	4 (19%)	9 (43%)	2 (10%)
Team	9 (43%)	4 (19%)	0 (0%)
Organization	8 (38%)	2 (10%)	9 (43%)

#### Leaders and leadership styles examined

Few studies described the assigned or intended role of the first-level leader in implementation (29%). Descriptions of first-level leaders’ roles were often vague, with the richest description stating, “leaders were required to manage the new programs, provide supervision, and also arrange financing for the program” [95].

A glossary of leadership constructs examined is included in Table 5. Most studies examined a general style of leadership (76%). Transformational leadership (67%) was the most common style of general leadership examined, followed by transactional (29%) and passive-avoidant (19%) leadership. Fifty-seven percent of studies examined leadership that strategically focused on implementation. These included implementation leadership (52%), behaviors described in the theory of middle managers’ role in implementation (29%) [48], and leaders acting as EBP champions (10%). While general leadership was most often measured by quantitative surveys, strategic implementation leadership was primarily measured through qualitative interviews. Additionally, studies described leader behaviors (26%) and characteristics (5%) that could not be otherwise categorized into existing leadership theories.

**Table 5:** Glossary of leadership constructs

Term	Definition
First-level leadership [8–10, 18]	A process of intentional efforts, by an individual who directly supervises frontline employees who do not manage others, to motivate, influence, and enable a person or group of people with the aim of impacting group or organizational outcomes
Transformational leadership [41]	Inspiring and motivating employees to perform beyond expectations
Inspirational motivation	Possessing a shared vision and high expectations that inspire and motivate others
Idealized influence	Embodying the values and behaviors to fulfill this vision
Intellectual stimulation	Challenging others to rethink ways they perform their duties and soliciting their ideas
Individualized consideration	Attending to the individual needs and feelings of employees
Transactional leadership [41]	Relying on reinforcement and exchanges to promote performance on tasks that are part of one’s role
Contingent reward	Assigning and setting reward contingencies for fulfilling tasks
Management by exception–active	Actively identifying and addressing employee mistakes or performance shortcomings
Passive-avoidant leadership [42]	Avoiding making decisions and/or managing employees
Management by exception–passive	Passively waiting for errors and issues and then addressing them
Laissez-faire	Taking a “hands off” approach by altogether avoiding making decisions or managing employees
Implementation leadership [20]	Leading in ways that are intended to promote the implementation of EBP
Proactive	Anticipating and addressing implementation challenges
Knowledgeable	Possessing a deep understanding of EBP implementation
Perseverant	Being consistent, resolute, and responsive to EBP implementation
Supportive	Supporting providers’ EBP adoption and use
EBP champion [64]	Convincing others to accept the innovation through educating, advocating, building relationships and navigating boundaries
Effective organization	Coordinating employees and tasks to efficiently accomplish goals
Managing team relationships	Promoting group cohesion by attending to social dynamics between employees
Equitably distributing work	Dividing work tasks in a fair manner
Managing competing priorities	Attending to implementation tasks among competing priorities and workloads
Facilitating communication	Ensuring information is communicated across varying levels of an organization
EBP buy-in	Agreeing with or supporting an EBP and its implementation

*EBP* evidence-based practice

#### Inner-context factors

A glossary of inner-context factors examined is included in Table 6. Twelve studies (57%) examined the relationship between leadership and two of the five broad inner-context constructs: individual characteristics (38%) and organizational characteristics (38%). Individual characteristics that were specifically focused on EBPs (i.e., providers’ EBP attitudes and EBP knowledge) were more common than individual characteristics that were generally focused (i.e., providers’ burnout). Providers’ EBP attitudes was the most common inner-context outcome examined (24%). Organizational characteristics had a slight tendency to be generally focused (i.e., organizational climate and organizational culture) rather than strategically focused (i.e., implementation climate and innovation climate).

**Table 6:** Glossary of inner-context factors

Term	Definition
Organizational characteristics	Structures or processes that take place and/or exist in organizations that may influence implementation [29]
Implementation climate	The extent to which organizational members perceive that innovation use is expected, supported, and rewarded [56]
Innovation climate	The shared perception of the extent of an organizations’ openness to new innovations [57]
Organizational climate	“The shared meaning organizational members attach to the events, policies, practices, and procedures they experience and the behaviors they perceive being expected, rewarded and supported” [58]
Empowering climate	Organizational members perceptions of fairness, perceived opportunities for growth and advancement, and role clarity [59]
Demoralizing climate	Organizational members perceptions of depersonalization, emotional exhaustion, and role conflict [59]
Organizational culture	The norms and shared behavioral expectations within an organization [60]
Individual characteristics	Shared or unique characteristics of individuals (e.g., provider, supervisor, director) that influence implementation [29]
Burnout	A psychological syndrome characterized by emotional exhaustion, depersonalization, and reduced personal accomplishment [61]
Attitudes toward EBPs	Provider perceptions of the appeal of EBPs, requirements to adopt EBPs, openness to innovation, and perceived divergence between new and current practice [62]
Knowledge of EBPs	Provider familiarity, awareness, or understanding of EBPs

*EBP* evidence-based practice

#### Implementation outcomes

Eleven studies (52%) examined the relationship between leadership and an implementation outcome, including five studies (24%) that examined a specific implementation outcome as defined by Proctor and colleagues [63]. These included adoption (14%), fidelity (5%), and sustainment (5%). The remaining six studies (29%) examined “general implementation,” often encompassing multiple implementation outcomes.

### Relationships between leadership and inner-context outcomes

A summary of the quantitative direct and indirect relationships between leadership and inner-context and implementation outcomes is included in Table 7. Results from the qualitative synthesis are listed in Table 8. Most studies (43%) examined the relationship between general leadership and an inner-context outcome [45, 65, 81, 82, 84–86, 88, 92], and fewer studies examined the relationship between strategic implementation leadership and an inner-context outcome (24%) [15, 45, 65, 92, 97].

**Table 7:** Relationships between leadership and inner-context and implementation outcomes

Leadership style	*N* (%)	Dir	Inner-context outcomes	*N* (%)	Dir	Implementation outcomes
General leadership						
Transformational	1 (5%)	–	Burnout [85]	1 (5%)	+	Sustainment [83]
	5 (24%)	+/–	EBP attitudes^a^ [81, 82, 84, 86, 92]			
	1 (5%)	+/	EBP knowledge [92]			
	1 (5%)	+	Innovation climate^a^ [82]			
	2 (10%)	+	Organizational climate [84, 88]			
	2 (10%)	+ –	Organizational culture^a^ [85, 98]			
Transactional	1 (5%)	/	Burnout^c^ [85]	1 (5%)	/	Sustainment^d^ [83]
	1 (5%)	+	EBP attitudes^ab^ [81]			
	1 (5%)	/	Organizational culture^ac^ [85]			
Passive-avoidant	1 (5%)	/	Burnout [85]	1 (5%)	–	Sustainment^d^ [83]
	1 (5%)	–/	Organizational culture^a^ [85]			
Strategic leadership						
Implementation	2 (10%)	+/–	EBP Attitudes^a^ [15, 92]	2 (10%)	+/	Adoption [15, 97]
	1 (5%)	/	EBP Knowledge [92]			
	1 (5%)	+	Implementation Climate^a^ [97]			
	1 (5%)	+	Organizational Climate^a^ [45]			
Leader characteristics						
EBP attitudes				1 (5%)	+/	Adoption [89]
Readiness-for-change				1 (5%)	+/	Adoption [89]

+ Significant positive association between constructs

– Significant negative association between constructs

/ Non-significant association between constructs

+/ Significant positive association and a non-significant association between constructs

–/ Significant negative association and a non-significant association between constructs

+ – Significant positive and negative association between constructs

+/– Significant positive, significant negative, and non-significant between constructs

Statistical significance determined as *p* < .05. The direction of relationships can be mixed within a study if they report on subscales of a measure. We report only on total scores of a measure when available, otherwise, we report on subscale scores. Results reflect direct and indirect associations. When leader and provider scores were reported for the same measure, we report only the provider scores

^a^Reflects constructs that have been reframed to reflect the positive form of the construct (e.g., transformational leadership showing a negative association with demoralizing organizational climate is characterized as having a positive association with organizational climate)

^b^Measurement of transactional leadership included contingent reward, active management-by-exception, passive management-by-exception, and laissez-faire

^c^Measurement of transactional leadership included contingent reward, active management-by-exception, and passive management-by-exception

^d^Domains comprising transactional leadership measurement were not defined

**Table 8:** Barriers and facilitators of implementation outcomes and inner-context outcomes

	Barriers	*N* (%)^a^	Facilitators	*N* (%)^a^
Inner-context outcomes				
Implementation Climate	Strategic leadership	1 (5%)	General leadership	1 (5%)
	Low implementation leadership [65]	1 (5%)	High transformational leadership [65]	1 (5%)
	Low middle managers’ implementation role [65]	1 (5%)	Strategic leadership	1 (5%)
			High implementation leadership [65]	1 (5%)
			High middle managers’ implementation roles [65]	1 (5%)
Implementation outcomes				
Fidelity	General leadership	1 (5%)	General leadership	1 (5%)
	Low transformational leadership [90]	1 (5%)	High transformational leadership [90]	1 (5%)
	Behaviors	1 (5%)	High transactional leadership [90]	1 (5%)
	Inequitable workload distribution [90]	1 (5%)	Strategic leadership	1 (5%)
	Poor organization [90]	1 (5%)	High implementation leadership [90]	1 (5%)
	Poor management of team relations [90]	1 (5%)	High middle managers’ implementation roles [90]	1 (5%)
			Behaviors	1 (5%)
			Equitable workload distribution [90]	1 (5%)
			Good management of team relations [90]	1 (5%)
Implementation	General leadership	3 (14%)	General leadership	2 (10%)
	Low transactional leadership [87, 93]	2 (10%)	High transformational leadership [95, 96]	2 (10%)
	High passive-avoidant leadership [93, 96]	2 (10%)	Strategic leadership	3 (10%)
	Strategic leadership	3 (14%)	High implementation leadership [91, 94]	2 (10%)
	Low implementation leadership [91, 93, 96]	3 (14%)	High middle managers' implementation roles [91, 94]	2 (10%)
	Low middle managers’ implementation roles [93]	1 (5%)	High EBP champion [95]	1 (5%)
	Behaviors	3 (14%)	Characteristics	1 (5%)
	Poor management of competing priorities [93, 95]	2 (10%)	High EBP buy-in [68]	1 (5%)
	Poor communication [87]	1 (5%)		
	Characteristics	1 (5%)		
	Low EBP buy-in [91]	1 (5%)		
Sustainment			Strategic leadership	1 (5%)
			High implementation leadership [83]	1 (5%)
			High middle managers’ implementation roles [83]	1 (5%)
			High EBP champion [83]	1 (5%)

Transactional leadership only refers to the domain contingent reward

#### Individual characteristics

The relationship between transformational leadership and providers’ EBP attitudes was the most commonly examined relationship (24%) across all outcomes. Four studies showed some evidence of a positive relationship between transformational leadership and providers’ EBP attitudes [81, 82, 84, 86] and two supported a positive relationship between implementation leadership and EBP attitudes [15, 92]. However, these studies also reported mixed leadership–attitude relationships. One study found transformational leadership positively predicted provider attitudes in the context of EBP implementation, but was unrelated to EBP attitudes among providers delivering services-as-usual [82]. Another study found positive, negative, and non-significant relationships between general and strategic leadership and EBP attitudes [92]. It is notable that this study included several theoretically correlated predictors in their model (e.g., general and strategic leadership), which raises the question of whether multicollinearity may explain the negative relationships between leadership and EBP attitudes. *Individual consideration*, one of four domains of transformational leadership involving attention to individual needs and feelings of employees, was *negatively* related to EBP attitudes. With regard to implementation leadership, *proactive leadership*, involving anticipating and addressing implementation challenges, was positively related to EBP attitudes. Another domain *perseverant leadership*, which is consistent, resolute, and responsive to EBP implementation, was *negatively* related to EBP attitudes [92].

#### Organizational characteristics

While there were mixed relationships between leadership styles and organizational characteristics, three studies (14%) supported a positive relationship between either transformational or implementation leadership and organizational climate [45, 84, 99]. Among the qualitative studies, one study (5%) examined the relationship between leadership and an organizational characteristic [65]. High engagement in behaviors reflecting transformational leadership, implementation leadership, and middle managers’ role in implementation supported implementation climate. Low engagement in implementation leadership, specifically supportive leadership, and the middle managers’ role in implementation, hindered implementation climate.

#### Mediators of leadership and inner-context outcomes

Three studies (14%) examined the relationship between leadership and inner-context outcomes indirectly. The mediators examined included leader-provider relationship [82, 86], innovation climate [82], and organizational climate [84]. The strength of the leader-follower relationship mediated the relationship between transformational leadership and EBP attitudes [86] and innovation climate [82]. Organizational climate mediated the relationship between transformational leadership and EBP attitudes [84].

### Relationships between leadership and implementation outcomes

Eight studies (38%) examined the relationship between strategic implementation leadership and an implementation outcome, the majority (75%) of which were qualitative. The most studied relationships were the facilitating and hindering role of implementation leadership (19%) and middle managers’ role in implementation (14%) on general implementation. When leaders engaged in strategic implementation leadership, this facilitated general implementation and when leaders had an absence of strategic implementation leadership, this hindered general implementation. Two studies (10%) quantitatively examined the relationship between implementation leadership and adoption. One found a positive association [97], while the other did not [15]. No studies quantitatively examined the relationship between strategic implementation leadership and sustainment.

Six studies (29%) examined the relationship between general leadership and an implementation outcome [69, 83, 87, 93, 95, 96], the majority (83%) of which were also qualitative. The relationship between general leadership and general implementation was most often studied. The presence of transformational leadership facilitated general implementation (10%) [95, 96], while the presence of passive-avoidant leadership (10%) [93, 96], and absence of transactional leadership (10%) [87, 93] hindered general implementation. One study (5%) quantitatively examined the relationship between general leadership and sustainment [83]. They found that transformational leadership was positively related to sustainment, transactional leadership was non-significantly related to sustainment, and passive-avoidant leadership was negatively associated with sustainment [83].

#### Mediators of leadership and implementation outcomes

In the two studies (10%) that examined the indirect relationship between leadership and an implementation outcome, one found that implementation climate mediated the relationship between implementation leadership and adoption [97], while the other did not find support for providers’ EBP attitudes mediating the relationship between implementation leadership and adoption [15].

### Combined first-level and higher-level leadership

The most notable differences between studies that combined perceptions of first-level leadership with higher leadership levels relative to studies that *only* examined first-level leadership were (1) study method, (2) styles of leadership examined, and (3) outcomes examined. Studies with mixed leadership tended to be qualitative (54%), more often examined strategic implementation leadership (77%), and more frequently examined a specific implementation outcome (46%) or general implementation (46%). Of note, 31% of these studies examined the relationship between leadership and sustainment, relative to only 5% in the first-level leadership only sample. These studies primarily provide additional support for the facilitating role of strategic implementation leadership, and to a lesser extent positively framed general leadership, in supporting implementation outcomes (see Table 12 in additional file 3).

### Conceptual overlap in leader behaviors

Of all coded leader behaviors, 22% were categorized into more than one leadership style or theory. Among these overlapping codes, the majority came in two specific combinations. This included overlap in behaviors described in (1) the theories of implementation leadership and middle managers’ role in implementation (58%) and (2) transformational leadership and theory of middle managers’ role in implementation (17%).

The overlap in the theories of implementation leadership and middle managers’ role in implementation occurred most (29%) between two behaviors: (1) mediating between strategy and day-to-day activities (*mediating*) and (2) proactive leadership (*proactive*). Notably, no implementation leadership codes overlapped with the middle manager role termed *selling innovation implementation*.

## Discussion

This scoping review synthesized research examining the relationship between first-level leadership and inner-context and implementation outcomes in the context of behavioral health service delivery. Studies primarily relied on observational designs and were often cross-sectional. Studies most often examined general forms of first-level leadership, namely transformational leadership. Strategic implementation leadership was also the focus of over half of studies. Nearly equal attention was given to inner-context and implementation outcomes, and inner-context outcomes centered on organizational and individual characteristics. The greatest concentration of studies examined first-level leadership in relation to providers’ EBP attitudes and general implementation. Our synthesis revealed moderate conceptual overlap of leadership behaviors described in theoretical frameworks of strategic implementation leadership.

While the scope of the literature was broad, patterns emerged in the forms of leadership and outcomes studied across qualitative and quantitative investigations. Strategic leadership was more often the focus in qualitative studies, while quantitative studies commonly focused on general leadership. This likely reflects that quantitative measures of strategic implementation leadership have only recently been developed [46] while measures of general leadership have long been available [100]. Among outcomes studied, there was nearly equal representation of inner-context and implementation outcomes. However, over half of the literature informing our understanding of the relationship between first-level leadership and implementation measured general implementation, limiting our knowledge of the *precise* domains of implementation that are influenced by leadership. Further, of the five studies that measured a specific implementation outcome, three were self-reported. One notable exception was the use of independent observation to measure fidelity.

Few leadership-outcome relationships were examined across multiple studies; however, one exception was the focus on providers’ EBP attitudes. Studies primarily found positive associations in the direct and indirect relationships between transformational leadership and providers’ EBP attitudes, although there were exceptions. This finding aligns with a comprehensive synthesis of studies across occupational settings and leadership levels that support transformational leaderships’ positive relation to employee attitudes [42]. In the implementation literature, theory suggests positive EBP attitudes are a necessary precursor to the EBP adoption decision [101], yet the evidence linking providers’ attitudes and behavior is mixed. Studies conflict in their conclusions regarding providers’ EBP attitudes and EBP implementation-related behaviors, with some finding positive associations [102, 103] and others—including the most rigorous among these [104]—finding mixed or no associations [104–106]. What remains to be studied is whether the relationship between transformational leadership and providers’ EBP attitudes has implications for providers’ implementation *behaviors*. For instance, attitudes may modify the relationship between transformational leadership and adoption, such that transformational leadership may only predict adoption when providers have positive EBP attitudes.

Theory and research suggest general climates form a foundation for strategic climates, and these strategic climates function as a more proximal predictor of individual and organizational performance [58]. Ample evidence suggests that strategic climates mediate the relationship between leadership and employee performance [107, 108]. Further, strategic leadership has shown a stronger effect on strategic climate than general high-performance leadership [107]. Theory within implementation also posits that leadership influences organizational characteristics, including culture and climate, which then shape provider adoption and sustainment of EBPs [4, 5, 109]. While the relationship between first-level leadership and various organizational characteristics was a primary focus of the reviewed studies, variability in the specific outcomes measured and inconsistent findings offer limited conclusions about the nature of this relationship. Studies revealed some consistent, yet sparse, support for general and strategic implementation leadership positively relating to general [84, 88, 98] and strategic organizational climates [82, 97]. Studies demonstrated that general and strategic organizational climates mediated the relationship between positively framed leadership and other inner-context outcomes; however, only one study examined its mediating role on an implementation outcome. Williams and colleagues [97] provide initial evidence that the relationship between implementation leadership and EBP adoption is mediated by implementation climate. Extending from organizational theory and research, more evidence is needed to determine (1) whether strategic implementation leadership is associated with better strategic implementation climate, (2) whether general leadership and general climate are necessary precursors for positive implementation leadership and implementation climate, and (3) the mediating role of organizational characteristics in the relationship between leadership and implementation *outcomes*.

Leadership has shown a positive relationship with employees’ performance outcomes across industries [110]; however, the evidence linking leadership to implementation outcomes in behavioral health remains sparse. The qualitative evidence in this review indicates that first-level leadership, particularly strategic implementation leadership, facilitated general implementation. Yet it provides limited evidence regarding the influence of leadership behaviors on *specific* implementation outcomes. The studies involving mixed levels of leadership provide further support for the facilitating impact of strategic implementation leadership on implementation outcomes, although they preclude us from fully attributing that effect to first-level leadership. There were notably few studies that quantitatively examined the direct or indirect relationship between first-level leadership and implementation outcomes, limiting generalizations about these relationships. Findings suggest that implementation leadership and leader characteristics may support EBP adoption [89, 97], although additional research is needed as conflicting results exist [15]. Furthermore, measurement of EBP adoption has relied solely on provider report, dampening confidence in estimates of the leadership-adoption relationship [111].

### Broad strengths

The reviewed studies had a number of strengths. Notably, studies drew from the organizational behavior literature to guide conceptualizations of leadership styles and organizational contextual factors that influence providers’ EBP implementation. Use of validated and psychometrically sound measures of leadership and inner-context outcomes was a particular strength, especially given the lack of established implementation measures in the broader literature [112]. Studies tended to analyze perceptions of first-level leadership and implementation outcomes at the team and organization level, respectively. This aligns with calls for leadership research to move beyond studying how leadership affects individual-level processes [17]. Nonetheless, they also had a number of limitations that inform our future recommendations.

### Recommendation 1: improve methodological quality

Measurement and study design issues threaten progress in understanding how first-level leadership relates to implementation. Studies relied heavily on self-report measures, often by a single reporter. Most of the studies that quantitatively measured an implementation outcome were based on self-report. These measurement issues have produced overestimated parameters for leadership–outcome relationships [42, 111], and may contribute to inconsistent or conflicting results. While self-report may be most appropriate for many inner-context outcomes conceptualized as latent constructs, implementation outcomes are often directly observable. To address common method variance, studies should rely on multiple respondents for self-reported outcomes, such as measuring aggregate perceptions of organizational characteristics from all available employees, not only those reporting on leadership. When common methods are unavoidable, studies should follow procedural and statistical remedies to reduce bias (see Podsakoff and colleagues [113]). Studies should use direct observation of practice and record review to measure implementation outcomes such as adoption, fidelity and sustainment.

Two-thirds of studies that examined the indirect relationship between leadership and outcomes did so using a cross-sectional observational design, likely producing biased estimates [42]. While comprehensive recommendations for mediation analysis are described elsewhere [114, 115], we highlight those that are most feasible and relevant. Studies should include (1) more than two assessment points to establish temporal precedence [115]; (2) variables measured at all time points to assess for the reciprocity of mediating effects (e.g., changes in implementation climate lead to changes in EBP adoption and not vice versa) [114]; and (3) measurement should occur when changes in the mediator are expected to cause changes in the inner-context or implementation outcome.

### Recommendation 2: address conceptual overlap in first-level leadership

There have been calls for clarification of the converging and unique contributions of the theory of implementation leadership [46] and the theory of middle managers’ role in implementation [47], noting similarities in the behaviors described [97]. Our synthesis supports the claim that these theories are partially overlapping and suggests that middle managers’ implementation role of *mediating between strategy and day*-*to*-*day activities* converges with *proactive leadership*. These concepts overlap in their focus on removing implementation obstacles, addressing barriers to implementation, and supporting a strategy or plan for implementation. Our findings also suggest that these theories diverge with regard to middle managers’ implementation role of *selling the innovation*, a role defined as “presenting, convincing, and encouraging stakeholders to participate in implementation of an innovation” [49] (p. 9). In our synthesis, *selling the innovation* emerged as one of the most common ways that first-level leadership facilitated implementation, suggesting that it is a unique and important leadership behavior. Future research should examine the consistent and divergent aspects of these theories. For instance, template analysis could be used to explore this overlap, as was recently conducted to examine compatibility of the Ottawa Model of Implementation Leadership and the Implementation Leadership Scale [116].

### Recommendation 3: greater focus on implementation outcomes

One notable gap was the limited focus on specific implementation outcomes. While our qualitative synthesis suggests strategic implementation leadership and general leadership facilitates *general* implementation, the imprecise measurement limits our understanding of which outcomes they impact. Given their direct role in guiding treatment delivery and close relationship with providers [117], first-level leadership likely influences implementation outcomes that reflect providers’ EBP perceptions (e.g., acceptability) and their behaviors (e.g., adoption). Studies failed to examine implementation outcomes reflecting provider perceptions; however, these are likely related to the behavioral implementation outcomes [63]. For instance, the middle managers’ implementation roles *mediating* and *selling* may improve providers’ perceptions of EBP appropriateness, which may increase adoption. The literature would benefit from an increased focus on a broader set of implementation outcomes.

### Recommendation 4: examine how first-level leadership shapes team-level outcomes

The broader leadership literature has been limited by a dearth of studies examining how leadership impacts team-level outcomes [17]. In behavioral health, front-line employees often interact with first-level leaders as a team and deliver services as a team. For instance, a majority of providers in community mental health receive group-based supervision [117] and providers often use a team-approach to treatment delivery such group-based treatments [118] and team approaches for treating high-risk clients and severe mental illness [35, 119]. Future research should explore whether first-level leadership affects inner-context and implementation outcomes at the team-level.

### Recommendation 5: explore conditions under which leadership shapes implementation

Implementation is complex and highly context-dependent [120]. The inconsistencies in some leadership-outcome relationships may, in part, result from contextual factors that moderate these relationships. Studies of moderators were rare, yet two studies found that the influence of leadership on inner-context outcomes depended on whether an organization was actively implementing an innovation [82] and the degree of organizational stress [70]. The limited focus on moderators may miss characteristics of the organization, leader, provider, and EBP on which leadership-outcome relationship may depend. Leader characteristics, including EBP attitudes [89], EBP perceptions [71], and EBP buy-in [72, 91] that were related to implementation outcomes may moderate the relationship between leadership and implementation outcomes. The leadership literature points to possible provider-level moderators, such as self-efficacy [121], innovativeness [122], job tenure [123], and congruence of work with one’s values [123]. Future studies should explore moderators of leadership and inner-context and implementation outcomes. This will begin to address the question of, *under what circumstances does first*-*level leadership impact inner*-*context and implementation outcomes*?

### Limitations

Findings should be considered within the context of several limitations. First, we only included peer-reviewed empirical studies. As such, we excluded non-peer-reviewed studies and study protocols. Recent protocols show promising methods that will likely advance our understanding of first-level leadership and implementation [20, 124]. Further, while the inclusion of diverse study designs was most appropriate to address our aims, this precluded determinations about the validity of findings or reasons for discrepant findings in our sample. While search terms were developed to capture studies from diverse settings, most studies were conducted in the USA which may introduce bias.

## Conclusions

This review offers a synthesis of the current state of the literature examining how first-level leadership shapes implementation. The findings suggest that the impact of leadership in shaping inner-context outcomes, most notably providers’ EBP attitudes and the organizational context, has been a primary focus in quantitative analyses. In contrast, the facilitating and hindering role of strategic implementation leadership on general implementation have comprised much of the qualitative examinations. Our synthesis documents an important conceptual overlap in the theories of implementation leadership and middle managers’ role in implementation that should be disentangled, as these forms of strategic leadership appear to hold promise in supporting EBP implementation. We identified gaps in the literature and provide recommendations to advance our understanding of the relationship between first-level leadership and inner-context and implementation outcomes.

## Supplementary Information

**Additional File 1.** Preferred Reporting Items for Systematic reviews and Meta-Analyses extension for Scoping Reviews (PRISMA-ScR) Checklist.

**Additional File 2.** Literature Search Terms.

**Additional File 3.** Mixed-level Leadership Results.

## Data Availability

All articles included in this scoping review are publicly available. The datasets used and/or analyzed during the current study are available from the corresponding author on reasonable request.

## Abbreviations

A: Adoption
AH: Addiction health agencies
Bar: Barriers of implementation
CMH: Child mental health agencies
CW: Child welfare
DemoCli: Demoralizing climate
EBP: Evidence-based practice
EBPA: Evidence-based practice attitudes
EmpCli: Empowering climate
EPIS: Exploration, Preparation, Implementation, and Sustainment
Exp: Experimental
F: Fidelity
Fac: Facilitators of implementation
GI: General implementation
H: Hospital
I: Implementation
IC: Individual characteristics
IPP: Implementation policies and practices
IL: Implementation leadership
ImpCli: Implementation climate
InnCli: Innovation climate
L: Leadership
LB: Leader behaviors
LC: Leader characteristics
LfL: Laissez-faire leadership
LMX: Leader-member exchange
MBE: Management by exception
MH: Mental health agencies
Mul: Multiple phases-unspecified
N/A: Not applicable
NI: No active implementation
NR: Not reported
Obs: Observational
OC: Organizational characteristics
OrgCli: Organizational climate
OrgCul: Organizational culture
OSP: Organizational staffing processes
Oth: Other
P: Preparation
PaL: Passive-avoidant leadership
PRISMA-ScR: Preferred Reporting Items for Systematic Reviews and Meta-Analyses Extension for Scoping Reviews
Quas: Quasi-experimental
SMH: School-based mental health
Sus: Sustainment
TfL: Transformational leadership
TrC: Transactional culture
TrL: Transactional leadership
VA: Veteran’s Affairs Integrated Service Networks
