# Supplementary material for: The relationship between first-level leadership and inner-context and implementation outcomes in behavioral health: a scoping review
Source: Implement Sci. 2021 Jul 6;16:69. doi: 10.1186/s13012-021-01104-4 (PMC8259113; doi:10.1186/s13012-021-01104-4)
Supplement: Supplementary file 2 — Additional File 2. Literature Search Terms. [file 13012_2021_1104_MOESM2_ESM.docx]

**Additional File 2**

**Literature Search Terms**

(implement [tiab] OR implemented [tiab] OR implementer [tiab] OR implementation [tiab] OR adoption [tiab] OR disseminate [tiab] OR disseminated [tiab] OR dissemination [tiab] OR "knowledge translation" [tiab] OR "quality improvement" [tiab] OR "inner context" [tiab] OR "inner contextual" [tiab] OR "inner setting" [tiab] OR diffusion of innovation [MeSH Terms] OR organizational innovation [MeSH Terms])

AND

(leader [tiab] OR leaders [tiab] OR leadership [tiab] OR leadership [MeSH Terms] OR supervise [tiab] OR supervisor [tiab] OR supervision [tiab] OR manage [tiab] OR manager [tiab]OR managers [tiab] OR management [tiab])

AND

("behavior health" [tiab] OR "behavior healthcare" [tiab] OR "behavioral health" [tiab] OR "behavioral healthcare" [tiab] OR "behavioural health" [tiab] OR "behavioural healthcare" [tiab] OR "behaviour health" [tiab] OR "behaviour healthcare" [tiab] OR "mental health" [tiab] OR "mental healthcare" [tiab] OR addiction health [tiab] OR addiction healthcare [tiab] OR Mental Health Services/organization and administration [MeSH Terms] Mental Health Services [MeSH Terms])

AND

(evidence based [tiab] AND practice [tiab] OR practices [tiab] OR treatment [tiab] OR treatments [tiab] OR intervention [tiab] OR interventions [tiab] OR empirically supported [tiab] AND treatment [tiab] OR treatments [tiab] OR innovation [tiab] OR innovations [tiab] OR "best practice" [tiab] OR "best practices" [tiab] OR evidence based practice [MeSH Terms])
