# Supplementary material for: The relationship between first-level leadership and inner-context and implementation outcomes in behavioral health: a scoping review
Source: Implement Sci. 2021 Jul 6;16:69. doi: 10.1186/s13012-021-01104-4 (PMC8259113; doi:10.1186/s13012-021-01104-4)
Supplement: Supplementary file 3 — Additional File 3. Mixed-level Leadership Results. [file 13012_2021_1104_MOESM3_ESM.docx]

**Additional File 3**

Mixed-level Leadership Results

**Table 9** Mixed-level leadership study characteristics (N = 13)

| **Characteristics** | ***N* (%)** |
| --- | --- |
| Setting^a^ |  |
| Mental health agencies | 10 (77%) |
| Substance use agencies | 2 (15%) |
| Hospital | 3 (23%) |
| Other | 1 (8%) |
| Country |  |
| Australia | 1 (8%) |
| Canada | 1 (8%) |
| United Kingdom | 2 (15%) |
| United States | 9 (69%) |
| Design |  |
| Observational | 13 (100%) |
| Method |  |
| Quantitative | 2 (15%) |
| Qualitative | 7 (54%) |
| Mixed | 4 (31%) |
| Data source^a^ |  |
| Survey | 6 (46%) |
| Interview | 9 (69%) |
| Focus groups | 4 (31%) |
| Field notes | 4 (31%) |
| Record reviews | 1 (8%) |
| Used a leadership questionnaire | 3 (23%) |
| Used a validated questionnaire (*n* = 3) | 1 (33%) |
| Used an unvalidated questionnaire (*n* = 3) | 2 (67%) |
| Used an inner-context and/or implementation outcome questionnaire | 6 (29%) |
| Used a validated questionnaire (*n* = 6) 2 (33%) | |
| Used a partially validated questionnaire (*n* = 6) | 1 (17%) |
| Used an unvalidated questionnaire (*n* = 6) | 3 (50%) |
| Implemented a clinical innovation | 13 (100%) |
| Phase of implementation^a^ |  |
| Implementation | 9 (69%) |
| Sustainment | 3 (23%) |
| No active implementation | 1 (8%) |
| Not reported | 1 (8%) |
| Leadership examined^a^ |  |
| General leadership | 5 (38%) |
| Transformational leadership | 4 (31%) |
| Transactional leadership | 1 (8%) |
| Passive-avoidant leadership | 1 (8%) |
| Strategic leadership | 10 (77%) |
| Implementation leadership | 9 (69%) |
| Middle-managers’ implementation roles^b^ | 8 (62%) |
| Leader behaviors^b^ *(uncategorized)* | 4 (31%) |
| Leader characteristics^b^ *(uncategorized)* | 2 (15%) |
| Inner-context outcomes^a^ | 1 (8%) |
| Organizational characteristics^a^ | 1 (8%) |
| Organizational climate | 1 (8%) |
| Organizational staffing procedures | 1 (8%) |
| Turnover | 1 (8%) |
| Individual characteristics^a^ | 1 (8%) |
| Turnover intentions | 1 (8%) |
| Implementation outcomes^a^ | 5 (24%) |
| Adoption | 2 (15%) |
| Fidelity | 1 (8%) |
| General implementation | 6 (46%) |
| Sustainment | 4 (31%) |
| *N* = 13 unless specified otherwise. ^a^Responses are not mutually exclusive; ^b^The studies reporting these leadership styles and behaviors were all qualitative studies | |

**Table 10** Summary of studies with first-level and mixed levels of leadership (N = 13)

| **First**  **author** | **Leadership**  **style(s)** | **Inner-context outcomes** | **IS**  **outcomes** | **Design** | **Setting** | **IS**  **Phase** | **Key leadership findings** |
| --- | --- | --- | --- | --- | --- | --- | --- |
| Aarons et al., 2011 | TfL | OC, OSP | N/a | Obs | MH, AH, Oth | NI | The relation between TfL and providers' turnover intention was mediated by organizational OrgCli. More positive TfL positively predicted an EmpCli which in turn predicted lower provider turnover intentions. |
| Brooks et al., 2011 | LB, LC | N/a | GI | Obs | MH | I | Leader resistance and supporting the status quo was a barrier to implementation. Sustained leader ‘buy-in’ or support and willingness to solve problems of resistance as they arose facilitated implementation. |
| Byron et al., 2015 | LB | N/a | GI | Obs | H | I | Leaders’ insufficient efforts to gain staff's EBP buy-in was a barrier to implementation. Leaders acting as change agents through networking to address leadership and staff needs, having experience and familiarity with the intervention evidence, articulating the added value of the intervention by appealing to organizational values, and acting as a role model all facilitated implementation. Leaders also created buy-in by engaging employees and discussing the value of the intervention. |
| Einfeld et al., 2002 | LB | N/a | Sus | Obs | CMH, MH, H | I, Sus | Findings did not support an association between management directives, including overall leader support for the intervention, and sustainment of services. |
| Fearing et al., 2014 | LB | N/a | GI | Obs | CMH | I | Managers’ competing workload and unclear clinical supervision guidelines and structure were barriers to implementation. |
| Ford et al., 2011 | LB, LC | N/a | Sus | Obs | H | Sus | Low leadership support and leader resistance to change were barriers to implementation. Lack of leader involvement was a barrier to sustainability. Strongly involved leadership (e.g., monitoring activities, sharing providers' issues at manager meetings) facilitated implementation. |
| Guerrero et al., 2016 | LB, LC | N/a | GI | Obs | AH | NR | Leaders facilitated implementation by: demonstrating knowledge; proactively facilitating implementation; proactively creating a climate conducive to implementation; supporting change through individualized connections; supporting change through transactions; and persevering through problem-solving. |
| Nadeem et al., 2018 | LB | N/a | A | Obs | SMH | I | Leaderships' lack of involvement in the project after initial training was more common in sites that did not make it to the implementation phase. Leader participation in the decision-making process during the engagement phase, leadership that guided implementation and provided resources and leadership support for investing time in implementation were more common in sites who implemented. |
| Rodriguez et al., 2018 | LC | N/a | Sus | Obs | CMH | Sus | Leaders’ more favorable perceptions of a practice increased the odds of practice sustainment. |
| Sommerfeld et al., 2019 | LB | N/a | A, Sus | Obs | MH | I | Multilevel leadership was identified as the second most important dimension in impacting intervention implementation and sustainability. Multilevel LBs included: communicating about intervention importance and garnering intervention buy-in and support. |
| Stein et al., 2013 | LB | N/a | GI | Obs | MH | I | Lack of a forum for troubleshooting with leaders was a barrier to implementation. Built-in support mechanisms led by leaders facilitated implementation. |
| Whitley et al., 2009 | LB, LC | N/a | F | Obs | MH | I | Leadership void was the greatest barrier to EBP F, while strong and active leadership was the strongest facilitator. Other leader barriers to F included: not proactively engaging trainers or incorporating their suggestions; disinterest in learning new practices and perceiving the intervention as a burden; being unconvinced about the intervention. Leader facilitators of F included: creative synergy between leadership at different levels; being convinced of the treatment desirability and efficacy. |
| Xandis & Gumley, 2019 | LB | N/a | GI | Obs | MH | I | Leaders' enthusiasm, having specialized training in the intervention, proactively seeking referrals, and having central involvement in implementation facilitated implementation. Leaders' limited awareness of the intervention and how it differed from other interventions was a barrier to implementation. |

*A* adoption, *AH* addiction health agencies, *CMH* child mental health agencies, *EBP* evidence-based practice, *EmpCli* empowering climate, *F* fidelity, *GI*, general implementation, *H* hospital, *I* implementation, *LB* leader behaviors, *LC* leader characteristics, *MH* mental health agencies, *N/A* not applicable, *NI* no active implementation, *NR* not reported, *Obs* observational, *OC* organizational characteristics, *OrgCli* organizational climate, *OSP* organizational staffing processes, *Oth* other, *SMH* school-based mental health, *Sus* sustainment, *TfL* transformational leadership

**Table 11** Relationships between mixed-level leadership and inner-context and implementation outcomes

| **Leadership style** | ***N (%)*** | **Dir.** | **Inner-context outcomes** | ***N (%)*** | **Dir.** | **Implementation outcomes** |
| --- | --- | --- | --- | --- | --- | --- |
| **General leadership** |  |  |  |  |  |  |
| Transformational | 1 (8%) | +/ | Organizational climate (100) |  |  |  |
|  | 1 (8%) | – | Turnover (100) ­ |  |  |  |
|  | 1 (8%) | – | Turnover intentions (100) |  |  |  |
| **Leader behaviors** |  |  |  |  |  |  |
| Management directives |  |  |  | 1 (8%) | / | Sustainment (117) |
| **Leader characteristics** |  |  |  |  |  |  |
| Practice perceptions |  |  |  | 1 (8%) | + | Sustainment (101) |

+ Significant positive association between constructs

– Significant negative association between constructs

/ Non-significant association between constructs

+/ Significant positive association and a non-significant association between constructs

*Notes*. Statistical significance was determined as *p* $<$ .05. The direction of relationships can be mixed within a study if they report on subscales of a measure. We report only on total scores of a measure when available, otherwise, we report on subscale scores. Results reflect direct and indirect associations. When leader and provider scores were reported for the same measure, we report only the provider scores.

**Table 12** Barriers and facilitators of implementation outcomes and inner-context outcomes among mixed-level leadership

|  | **Barriers** | ***N* (%)^a^** | **Facilitators** | ***N* (%)^a^** |
| --- | --- | --- | --- | --- |
| Adoption | General leadership | 1 (8%) | General leadership | 1 (8%) |
|  | High passive-pvoidant leadership (118) | 1 (8%) | Low passive-avoidant leadership (118) | 1 (8%) |
|  |  |  | Strategic leadership | 1 (8%) |
|  |  |  | High implementation leadership (118) | 1 (8%) |
|  |  |  | High middle managers’ implementation roles (118) | 1 (8%) |
| Fidelity | Strategic leadership | 1 (8%) | Behaviors | 1 (8%) |
|  | Low implementation leadership (102) | 1 (8%) | Good collaboration (102) | 1 (8%) |
|  | Characteristics | 1 (8%) | Characteristics | 1 (8%) |
|  | Low EBP buy-in (102) | 1 (8%) | High EBP buy-in (102) | 1 (8%) |
| Implementation | Strategic leadership | 5 (39%) | General leadership | 3 (23%) |
|  | Low implementation leadership (119–121) | 3 (23%) | High transformational leadership (74,121,122) | 3 (23%) |
|  | Low middle managers’ implementation Roles (74,116,119,120) | 4 (31%) | High transactional leadership (122) | 1 (5%) |
|  | Behaviors | 1 (8%) | Strategic leadership | 6 (46%) |
|  | Poor management of competing priorities (119) | 1 (8%) | High implementation leadership (74,116,120–122) | 5 (39%) |
|  |  |  | High middle managers’ implementation roles (74,121,123) | 3 (23%) |
|  |  |  | Behaviors | 1 (8%) |
|  |  |  | High implementation involvement (121) | 1 (8%) |
| Sustainment | Strategic leadership | 1 (8%) | Strategic leadership | 1 (8%) |
|  | Low implementation leadership (124) | 1 (8%) | High middle managers’ implementation roles (124) | 1 (8%) |
|  | Behaviors | 1 (8%) | Behavior | 1 (8%) |
|  | Low implementation involvement (124) | 1 (8%) | High implementation involvement (124) | 1 (8%) |
|  | Characteristics | 1 (8%) |  |  |
|  | Resistance (124) | 1 (8%) |  |  |

*Note.* Transactional leadership only refers to the domain contingent reward.
